# Older Adults-Friendly Multi-Modal Touch-Screen Platform Measures for Bilingual Cognitive Interventions

**DOI:** 10.1093/geroni/igaa057.2972

**Published:** 2020-12-16

**Authors:** W Quin Yow, Tharshini Lokanathan, Hui-Ching Chen

**Affiliations:** Singapore University of Technology & Design, Singapore, Singapore

## Abstract

There is an increasing interest in using touch-screen devices to conduct cognitive training and collect measurements of cognitive performance. However, older adults often have concerns such as anxiety about using these systems and poor comprehension of language instructions (Czaja & Lee, 2007). Given that Singapore is a multilingual society, we examined the deployment of an age-friendly multi-modal touch-screen platform (a game-based application on a tablet) in a cognitive intervention research. After modification of the platform to include features such as simplified instructions, multi-level prompts with a local accent, and four different instructional languages (including local dialects), participants were less reliant on the researchers and reported fewer difficulties in comprehending the instructions. The integrity and reliability of the data collected improved as a result. In sum, multilingual age-friendly touch-screen platform can be a novel yet effective method to study cognitive interventions in the Asian older adult populations.

